# Experimental evaluation of accuracy and efficiency of two control strategies for a novel foot commanded robotic laparoscope holders with surgeons

**DOI:** 10.1038/s41598-024-59338-3

**Published:** 2024-04-23

**Authors:** Yan-Jun Yang, Arvind Kumar N Vadivelu, Jessica Hepworth, Yongpeng Zeng, Charles H. C. Pilgrim, Dana Kulic, Elahe Abdi

**Affiliations:** 1 Department of Mechanical and Aerospace Engineering, Monash University, Clayton, VIC 3800 Australia; 2 The Department of Mechanical Engineering, The University of Melbourne, Parkville, VIC 3010 Australia; 3 Suite 29, Cabrini Medical Centre, Malvern, 3144 VIC Australia; 4 The Alfred Hospital, Malvern, VIC 3144 Australia; 5 Faculty of Medicine, Monash University, Clayton, VIC 3800 Australia

**Keywords:** Biomedical engineering, Surgery

## Abstract

The implementation of a laparoscope-holding robot in minimally invasive surgery enhances the efficiency and safety of the operation. However, the extra robot control task can increase the cognitive load on surgeons. A suitable interface may simplify the control task and reduce the surgeon load. Foot interfaces are commonly used for commanding laparoscope-holding robots, with two control strategies available: decoupled control permits only one Cartesian axis actuation, known as decoupled commands; hybrid control allows for both decoupled commands and multiple axes actuation, known as coupled commands. This paper aims to determine the optimal control strategy for foot interfaces by investigating two common assumptions in the literature: (1) Decoupled control is believed to result in better predictability of the final laparoscopic view orientation, and (2) Hybrid control has the efficiency advantage in laparoscope control. Our user study with 11 experienced and trainee surgeons shows that decoupled control has better predictability than hybrid control, while both approaches are equally efficient. In addition, using two surgery-like tasks in a simulator, users’ choice of decoupled and coupled commands is analysed based on their level of surgical experience and the nature of the movement. Results show that trainee surgeons tend to issue more commands than the more experienced participants. Single decoupled commands were frequently used in small view adjustments, while a mixture of coupled and decoupled commands was preferred in larger view adjustments. A guideline for foot interface control strategy selection is provided.

## Introduction

In conventional minimally invasive surgery (MIS), the surgical site is accessed through small incisions on the body. A laparoscope, operated by a camera handler assistant, is inserted to provide the surgeon with a view of the surgery site. A robotic laparoscope holder is applied in Robot-Assisted Minimally Invasive Surgery (RA-MIS) to replace the camera handler assistant in MIS. RA-MIS avoids issues such as surgeon-assistant communication inefficiency and assistant error, making it more efficient and safer than conventional MIS^1^.

The adoption of new technologies challenges the surgeon both cognitively and physically^2–4^. Performing open surgery has been shown to generate a very high cognitive workload, while MIS has even higher workload than open surgery since it demands additional cognitive skills, such as mentally correcting the laparoscope’s misorientation between visual information and horizon level^4,5^. In MIS, the surgeon’s work is usually divided into a primary hands-on operation, including the routine surgical procedures, and a separate decision-making task^6^. Surgeon’s cognitive resource is considered limited^6^. The cognitive resource is allocated to primary and secondary tasks according to the task demands, and if the resources are not consumed, spare cognitive perception helps the surgeon to have a better situation awareness and might improve their performance in advanced tasks^5^. However, the high cognitive workload in using new technologies and new additional tasks has been shown to affect surgical performance and can cause human error^3,7,8^. Excessive levels of mental workload may slow down decision-making and information processing^7,9^, and cause the surgeon to ignore potential hazards^6^. Therefore, when introducing a new surgical technique, it is highly desirable to reduce the surgeon’s cognitive load, making sure they have enough resources to cover all demands, maintain their primary task performance^6^, and have spare cognitive capacity for dealing with emergencies. In RA-MIS, in addition to the two conventional MIS tasks, the laparoscope control task becomes a source of extra cognitive load, demanding better workload management. Therefore, one of the challenges in RA-MIS is the implementation of a suitable human-robot interface to limit the cognitive load^10^. Recently, researchers have presented new algorithms in FI to tackle this challenge^11–13^.

The foot interface (FI) is commonly used in the laparoscope control task^14–16^. Apart from appropriate mechanical design, applying a suitable control strategy can reduce the surgeon’s workload. However, existing FI research mostly concentrates on delivering new mechanical designs with less focus on control. In this paper, we compare the two most widely adopted control strategies for mapping from FI to robot motions in this type of application: decoupled control and hybrid control. One main goal of this study is to look into a fundamental but key question that has not been investigated before: which is the best approach for the laparoscope manipulation task?

An interface needs to control at least four Degrees of Freedom (DoF) of a laparoscope to provide suitable focus and orientation of the target site view. Decoupled control actuates a single axis at a time, known as decoupled commands. Hybrid control allows separate actuation of each DoF as well as simultaneous actuation of multiple DoF, known as coupled commands. When proposing each of these designs, designers commonly make the following two assumptions:

- Decoupled control makes predicting the final view orientation of the laparoscope tip easier for the operator^14,17–19^.
- Hybrid control is more efficient compared to decoupled control^20–22^.

To the best of the authors’ knowledge, the above assumptions underlying the choice of control configuration have not been tested. This paper is the first study to validate the efficiency and predictability assumptions of the decoupled and hybrid approaches using a pure laparoscope manipulation task. A FI that is independent of most mechanical constraints was used for a fair comparison between the two control strategies. Eleven experienced surgeons and surgeon trainees were recruited, so the results and feedback were from the actual end users.

In addition, as another uninvestigated and related topic, this study explored how surgeons select and combine coupled and/or decoupled commands in surgery-like tasks considering the task requirement, different task phases and individual experience. This is referred to as the interface usage pattern. In this paper, the interface usage pattern and safety in two bi-manual surgery-like tasks are analysed.

**Figure 1:**
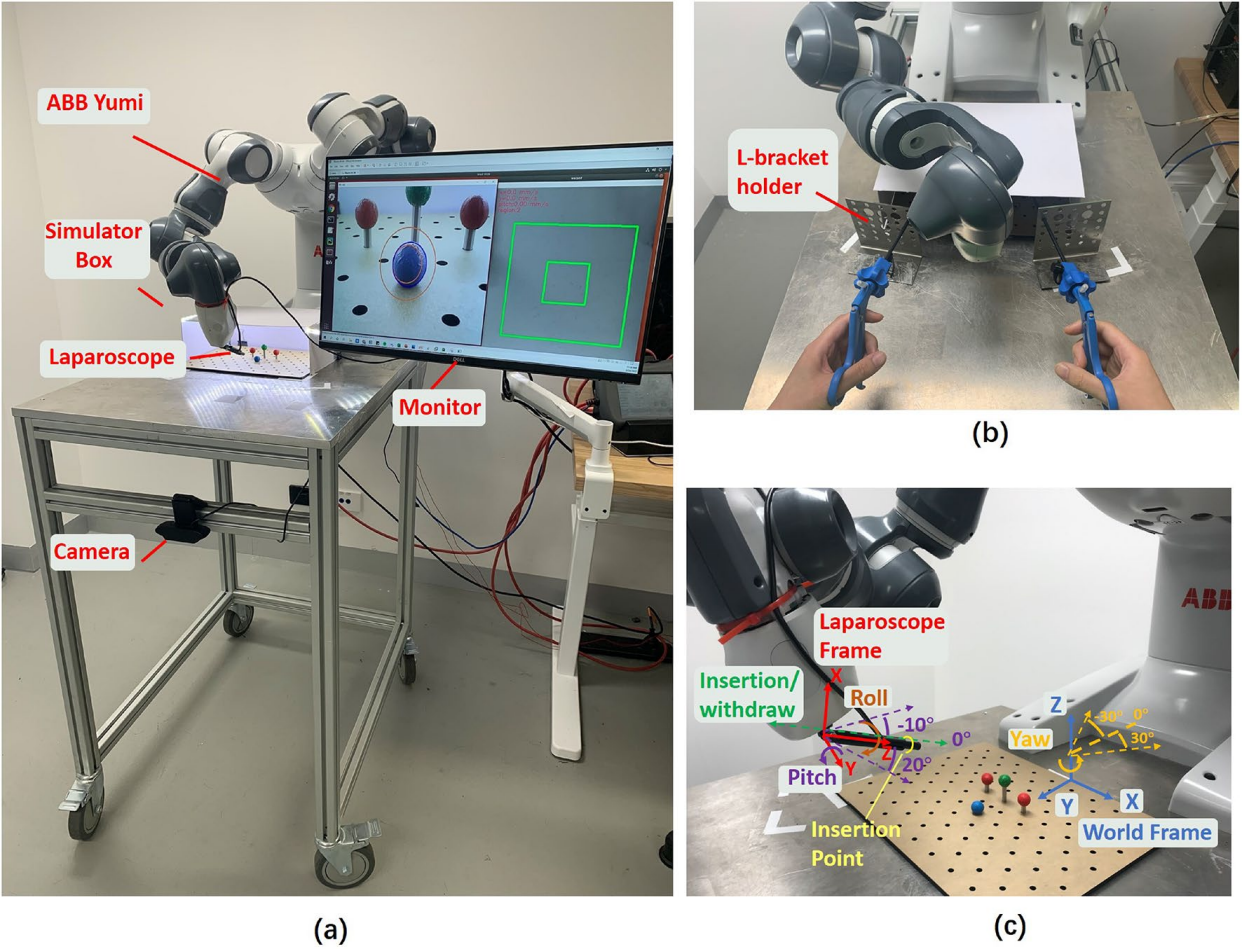
The laparoscope-holding robot used in the experiment: (**a**) The laparoscope-holding robot system consists of a YuMI robot, surgery simulator and a monitor. (**b**) The surgeon’s view when performing tasks. (**c**) Laparoscope required degrees of freedom. The world frame and local laparoscope frame are shown in blue and red, respectively. The four required DoF are also presented with respect to the laparoscope frame.

The rest of the paper is organised as follows: First, a system overview is provided, presenting the mechanical constraint-free FI, and the two studied control approaches. Then, the experimental platform setup and protocol are introduced, followed by the laparoscope control performance assessment criteria, FI usage pattern and safety evaluation methods. In the results section, the validation of the two control strategies’ assumptions is first described, followed by the usage pattern and safety analysis. The last two sections are the discussion and conclusion.

## System overview

The RA-MIS simulation layout (Fig. 1a) replicates the setup used in the operating room. The endoscope is inserted from the surgeon’s side, and both the endoscopic view and the FI overlook view are displayed on a monitor placed in front of the participants. The surgery simulator is a 300$\times$220$\times$100 mm^3^ open box covered by a board, so participants do not have a direct view of the operating site and must rely on the laparoscope view. An LED light is attached beneath the cover to provide sufficient and stable lighting. Two L-bracket holders with 6 mm hole openings (Fig. 1b) near the simulator are used to simulate trocar sleeves.

**Figure 2:**
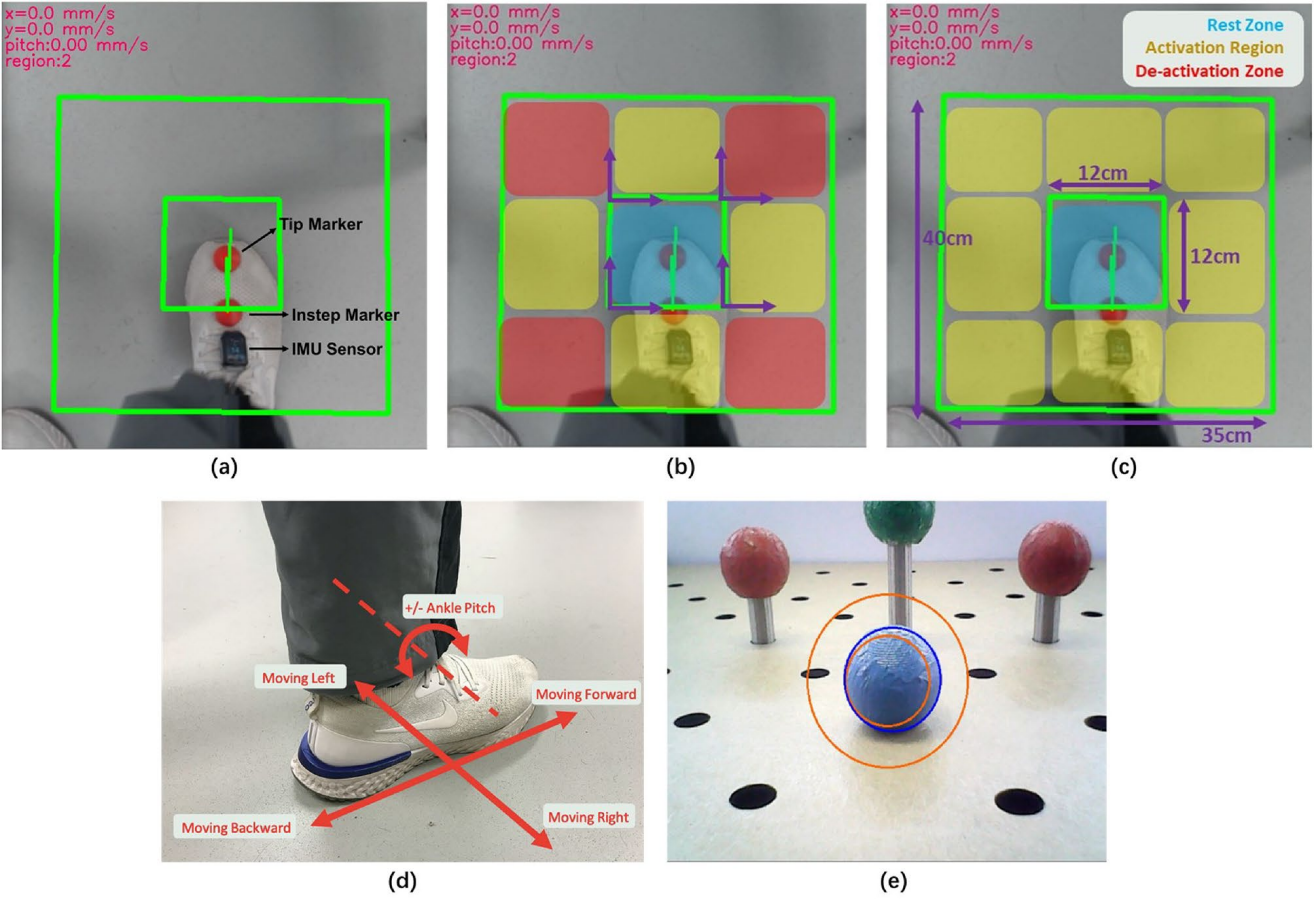
The foot interface design details: (**a**) FI overlook view shown on the monitor to the users. Two red markers are used for foot position/rotation tracking and an IMU sensor is used for ankle pitch tracking. (**b**) Foot map of decoupled control, local coordinates are shown in purple. Colour blocks are not shown to the surgeons. (**c**) Foot map of hybrid control. (**d**) Six foot basic commands: Moving forward/backward; left/right and bidirectional ankle pitch. (**e**) Laparoscopic view shown on the monitor to the users. The concentric circle mask is only used in the target aiming task.

### Robotic laparoscope holder

The foot-actuated robotic laparoscope holder is composed of an ABB IRB 14000 YuMI robot and a 720P DEPSTECH endoscope^23^ (Fig. 1a). A laparoscope needs at least four DoF (Fig. 1c), pitch, yaw, insertion/withdrawal, and roll to give a desired angle of view to the surgeon.

Pitch and yaw are used to adjust the translational movement of the laparoscopic view. Insertion/withdrawal relates to the view’s zoom factor, and roll controls the rotation of the image about an axis passing through the centre of the image. The laparoscope pitch motion is with respect to the local laparoscope frame (Fig. 1c), and the yaw motion is with respect to the predefined world frame Z-axis. Surgeons usually prefer the level of the laparoscopic view parallel to the ground level to ensure a comfortable orientation^24^. So adjusting roll is mostly needed when the surgeon corrects the laparoscopic view twist caused by the manual operation of the camera assistant. However, the level of the view is always parallel to the horizontal plane as the rotation of the laparoscope is constrained in the controller. Thus, roll is temporarily disabled in the system to reduce the user’s mental effort^24^.

The maximum controllable laparoscope pitch angle is between -10^∘^ to 20^∘^ and the maximum controllable yaw angle is between -30^∘^ to 30^∘^. The laparoscope insertion length is 6 cm due to the kinematic limitations of the YuMI robot. When the robot reaches the virtual workspace boundary, the control system stops the motion and displays a screen warning.

### Foot interface

Recently developed FIs have unique hardware design and/or algorithm optimization^14–16,25^ to ensure the dedicated control system runs smoothly and resists any noise or unintended commands. However, to compare the efficiency of the two control strategies and their ease of use, an intuitive and ergonomic FI independent of any specific control design is required.

#### Foot commands

To match the foot command and motion perceived from the laparoscopic view, moving the foot forward and backward controls pitch and moving the foot left and right maps to yaw control. Insertion/withdrawal is decided by the ankle lifting angle with respect to the reference plane initialised at the beginning of the experiment. The foot translation and ankle lifting are easier to perform and remember compared to a kick, shake, or shape trace^26^. Thus, the system has six basic foot-actions (Fig. 2d) for 3 DoF.

#### Input sensing

The FI (Fig. 2a) combines an environmental sensor (two 3D printed hemisphere markers with a radius equal to 1.5 cm and a 1080P Logitech C922 Pro web camera) and a wearable sensor (a Cometa WaveTrack IMU^27^) to detect the foot position/rotation and the ankle dorsiflexion/plantarflexion angle. The combination of wearable and external sensing allows the user to move their foot freely, naturally, and comfortably without any mechanical constraints^28^. The environment sensor detects the foot position and rotation along the axis perpendicular to the ground. The camera attached to the table (Fig. 1a) tracks the two markers attached to the user’s foot at the tip and instep. The tip marker is tracked continuously to represent the foot position. At the same time, the instep marker is used with the tip marker to calculate the ankle rotation angle. The distance between the two markers is also registered when the experiment starts as the reference to filter out any tracking error. The IMU sensor is attached to the user’s foot to detect the ankle pitch angle. The reference plane is initialised by averaging the user’s standing static data for two seconds at the beginning of the experiment. The accuracy of the IMU is between -0.5^∘^ to 0.8^∘^.

#### Decoupled control and Hybrid control

Decoupled control requires the surgeon to control a single DoF at a time. In contrast to decoupled control, hybrid control allows both actuation of single DoF commands and simultaneous multiple DoF commands. Therefore, the number of commands increased, including both the basic actions, three 2 DoF combinations (Pitch &Insertion, Yaw &Insertion, Pitch &Yaw) and one 3 DoF combination.

Foot commands are continuously tracked in a pre-defined region: The FI mapping area (Fig. 2b,c). It comprises an inner rest zone and an outer activation region. The activation region is divided into eight sub-sections composed of four diagonal regions and four unidirectional regions (Fig. 2c). Detection of the tip marker in the activation region actuates the robot. Inside the rest zone, the user can relax their foot in a natural gesture as any DoF except for insertion/withdrawal will be deactivated. Control will be paused if the tip marker is outside the activation region.

In decoupled control, a pitch command is issued if the tip marker is in the forward and backward unidirectional regions and a yaw command is sent to the robot if the marker is in the left and right unidirectional regions. The remaining four diagonal blocks are then identified as the non-activated regions. The robot performs no action if the tip marker is detected in these blocks.

Hybrid control allows both actuation of single DoF commands and simultaneous multiple DoF commands. In all unidirectional regions, foot commands include single DoF commands and the combination of a single DoF command plus insertion/withdrawal. In all the diagonal regions, foot commands include the dual translation DoF (pitch & yaw) command and the combination of the dual translation DoF command plus insertion/withdrawal. In the rest zone, insertion/withdrawal is still the only DoF that can be activated.

#### Workspace

The design of the size of the workspace was determined based on a size-evaluation pretest with one experienced surgeon and a trainee surgeon. The interface map size is 40 cm in length and 35 cm in width. It considers the torso-to-leg ratio^29^, and hip Flexion/Extension motion range^26^ of the two participants, and both of them could easily reach any target and use it for at least 15 minutes without apparent physical fatigue. The rest zone (length: 12 cm, width: 12 cm) dimensions were also chosen based on their suggestions.

Visual feedback is provided to allow the user to quickly and clearly identify the laparoscope’s moving state and locate their foot position based on common usage^30,31^. A command indicator window at the top left corner shows the current command and moving speed (Fig. 2a).

### Robot control

The robot uses velocity control with a 30 Hz control loop in this paper, and the control script is written in Python 2.7 to use the abb_robot_driver library^32^. In MIS, the laparoscope and surgical tools are inserted through small incisions into the patient’s body. Therefore, the laparoscope is constrained to move through and rotate about the incision point to prevent the incision port from tearing, known as the Remote Center of Motion (RCM) constraint. A programmable RCM constraint algorithm^33^ was implemented to control the endoscope holder end. The original algorithm was modified to use velocity input to replace the position input. The FI control integrates the RCM constraint, which means the laparoscope’s distal end will always move on the RCM sphere with the insertion point shown in Fig. 1c as the centre of the circle and the length from the insertion point to the laparoscope’s end as the radius. Based on the camera holding assistant and a surgeon’s suggestion, the speed range was from 0.02 rad/s to 0.08 rad/s for pitch and yaw, and 0 mm/s to 6 mm/s for insertion/withdrawal. The speed selection is a compromise between the reality of the surgery-like experiment setup and the performance limits of the robot.

The outputs from the FI are speeds in three DoF directions. The laparoscope pitch and yaw velocities positively correlate to the distance between the tip marker and the local coordinate that originates at the nearest vertex at the edge of the rest zone for the two control approaches (Fig. 2b). The DoF velocity is 0 if the tip marker is on the inner green edge (Fig. 2a), and the maximum velocity is issued if the tip marker is on the outer green edge. Insertion/withdrawal is decided by the ankle lifting angle with respect to the reference plane initialised at the beginning of the experiment. $-\,10^{\circ }$ to 10^∘^ is a dead zone to prevent disturbance. Any ankle pitch angle higher than the threshold (±18^∘^) will actuate the fastest speed. These two values were also determined according to the experienced and the trainee surgeon’s suggestion in the pretest.

In hybrid control, if no insertion/withdrawal command is detected, the resultant speed is that on the tangent surface of the RCM sphere. If the insertion speed is observed, the resultant speed sums the translational and insertion speeds. If the user intends to add only an additional insertion/withdrawal command while the tip marker is already in the outer activation region, it might lead to changes in the original translational DoF velocity output because the tip marker position can vary when the user adjusts their ankle pitch angle. Therefore, to interpret the user’s intention, two criteria are used: rotation angle change (with a threshold of 5^∘^) along the axis perpendicular to the ground and tip marker moving distance (with a threshold of 6 pixels). If both values are smaller than the thresholds, we assume the user only tends to add an insertion command, so the system adds the additional insertion/withdrawal based on the previous command. Otherwise, both the translation and insertion speeds would change.

## Experiment

We designed and conducted an experiment to test the two assumptions about the predictability and efficiency of the hybrid and decoupled control and to study the FI usage patterns. This project was approved by the Human Ethics Review Committee, Monash University (ID: 29291). The experiment was carried out by strictly following the guidelines of low-risk projects provided by the Human Ethics Review Committee, Monash University and informed consent was obtained from all participating surgeons.

To ensure that the assumptions are validated with the target user population, eleven surgeons (three females) with average age of 38.6 ± 6.1 years were recruited. Six of them are experienced surgeons, and five are still in training. All participants’ dominant foot is the right foot and all of them had experience with MIS, with at least 10 years experience for surgeons and an average of 5 years experience for surgeons in training. Seven participants had previously used FIs (foot pedals) for surgical use. Two experienced surgeons had experience with RA-MIS.

**Figure 3:**
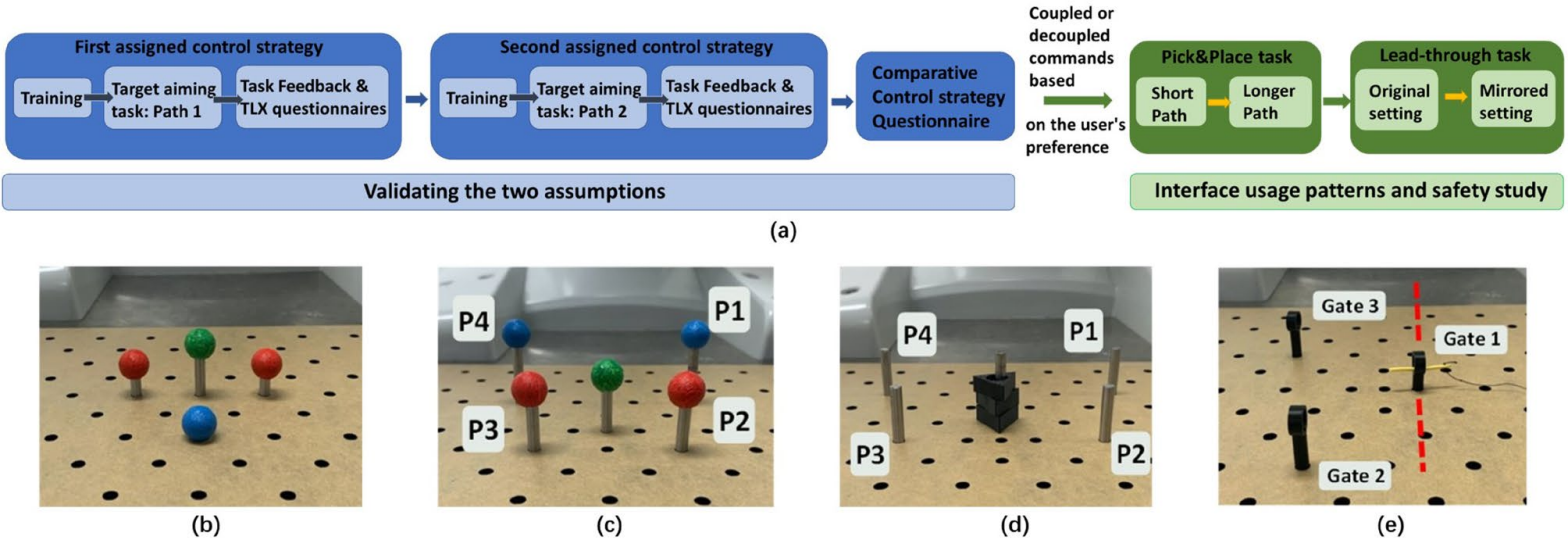
Experiment protocol: (**a**) Experiment flowchart (**b**) Training task setup. (**c**) Target aiming task setup. (**d**) Pick &Place task setup. (**e**) Lead-through task setup.

### Protocol

The experiment consists of two sets of tasks (Fig. 3a): the target aiming task where only laparoscope control was required, and the Pick &Place (hereby shortened as PP task) and Lead-through tasks (hereby shortened as LT task) where both laparoscope and surgical tool control was required. The target aiming task was performed first using each of the two control strategies to validate the two assumptions. Similar pure laparoscope control tasks are commonly used to test FIs with the proposed control strategy^14,15^. Then, in the two surgery-like tasks (PP and LT tasks), participants could freely use decoupled or coupled commands at any time. The two surgery-like tasks, both involving laparoscope-tool coordination skills and modified from MIS training courses^34^, were used to simulate a real operation and to study the FI usage pattern and safety when participants are simultaneously controlling the laparoscope and performing surgery-like tasks. In all three tasks, the participants were asked to perform the experimental tasks as fast as possible while trying to minimize operational errors.

Participants were asked to select the preferred foot to wear the FI equipment. They were then randomly assigned a control strategy to use first for the target aiming task and the performing sequence of the two surgery-like tasks.

**Training**: A board with four markers (Fig. 3b) was provided for five minutes of training, following a common protocol in similar research^20,35^. The participant was encouraged to explore every foot command and speed level and use the assigned control strategy to approach the markers. After this training phase, the target aiming task started.

**Target aiming task**: This task involves moving the laparoscope view to point to two targets in a given sequence. The setup consists of five poles, including one central pole, and four others arranged around the central pole, labelled P1, P2, P3 and P4 in a clockwise direction (Fig. 3c). The spherical object on top of the pole is the target. A concentric circle (inner radius: 60 pixel and outer radius: 110 pixels) is drawn on the endoscopic view for this task (Fig. 2e). The outer circle represents the region of interest and the optimal zoom factor is confined within the two circles. Thus, the contour of the target needs to be between the inner and outer circles. Once the target is between the concentric circles, the target outline will light up, and if the target remains in that area for 1 s, a “Move back to centre” or “Move back to corner” indication is displayed on the screen. The start position is right below the centre target. The participant is instructed to guide the laparoscopic view to the centre first, then aim to the next given target, and back to the centre to execute the same procedure for the following target. The time starts when the aim target sequence is shown on the screen and stops when the participant reaches one blue and one red target and aims back at the centre target. The basic task is repeated four times for two different aiming orders: Center-P1-Center-P3-Center and Center-P4-Center-P2-Center.

In this task, two poles (P1 &P4) are not visible from the starting pose, and two are close to the start point (P2 &P3). This setup replicates two situations: first, the target is close to the current site and small adjustments to the laparoscope view are needed. Second, the target is far from the laparoscopic centre view and large adjustments are needed, followed by more detailed aiming. After the first target aiming task, the same process was completed with the other control strategy.

**Pick & Place task**: The experiment setup (Fig. 3d) is similar to the target aiming task. The central pole has three triangular objects arranged in a stack. A surgical grasper and curved forceps are provided as the available tools. The participant is instructed to remove these triangular objects from the central pole using one tool, transfer the object to the other tool, and place the object on one of the surrounding poles, then repeat the task until all three objects are placed at the surrounding poles. At the centre pole, the participant can not see P4 and P1 and does not have a complete view of P3 and P2 before zooming out. The participant is required to keep the triangle object always in the view, which means the laparoscope adjustment is necessary during the task. The ready pose requires the participant to bring the tools in the endoscopic view. Time starts when the moving sequence is shown on the screen and finishes when the participant places the last object at the corresponding pole and withdraws tools out of view. The task is repeated four times with a shorter placement sequence (P1–P2–P3) first and then four times with a longer placement sequence (P1–P4–P2).

**Lead-through task**: A needle is placed in Gate 1 at the beginning (Fig. 3e). The participant is asked to use the provided surgical tools to hold the needle all the time and thread it through three circular gates (Gate2-Gate3-Gate1). The participant can choose the toolset of two needle holders or one needle holder and one curved forceps based on individual preference. Once the participant is in the ready pose, the time starts when the “start” command is shown on the screen and stops when the participant threads all gates and withdraws the tools out of view. This task is more difficult than the PP task as this task requires frequent movements of the laparoscope. It is repeated four times for the gates shown in Fig. 3e first, and then new gates are set by mirroring Gates 2 and 3 with respect to the red dotted line.

### Laparoscope control performance assessment

Four metrics were analysed: completion time, aiming accuracy, moving distance and the number of issued commands to compare the efficiency and predictability of the two control methods in the target aiming task and validate the two assumptions.

#### Efficiency

Efficiency is “the resources expended in relation to the accuracy and completeness with which users achieve the goal”^36^. In the target aiming task, the participant is expected to finish the task with relatively high accuracy and a short moving distance in a short time. Therefore, completion time, aiming accuracy and moving distance were considered when identifying the control method with better efficiency. The accuracy is presented as the average of the shortest distance from the target centre to the centre of the view in pixels when aiming at the three targets. The moving distance refers to the total laparoscope tip moving distance in the task.

#### Predictability

Predictability is evaluated based on the total number of issued commands in the target aiming task using the two testing scenarios (i.e. a close target and a further target). If the laparoscope is moving in a predictable manner, the participant should be able to aim at the target with fewer commands since the movement of the tool would match the operator’s expectations, easing the control of the laparoscope control.

#### Subjective assessment

The Comparative Control strategy Questionnaire, customized Task Feedback Questionnaire, and NASA Task Load Index Questionnaire^37^ were used in the experiment (Fig. 3a) mainly for the assumptions validation and control methods comparison.

The Comparative Control strategy Questionnaire compares the two control strategies’ predictability and efficiency in the target aiming task using single-choice questions at the end of the target aiming task. It also assesses the participants’ preference for a control strategy. The Task Feedback Questionnaire uses a five-point scale. It was used to assess the intuitiveness, usability and distraction of each control approach after the target aiming task.

The NASA Task Load Index Questionnaire is a subjective measurement of the participants’ perceived workload from six aspects, including effort, frustration, performance, mental, physical and temporal demand. Each factor’s raw scale is from 0 to 100 and has a weighted parameter from 0 to 5/15, with an interval of 1/15. The maximum weighted overall workload is 100. It is a method that enables participants to assess their experience with various human-machine interface systems.

### Interface usage pattern

The interface usage pattern study investigates how surgeons interact with the laparoscope-holding robot in the two surgery-like tasks. In this paper, the interface usage pattern is analyzed with respect to the motion patterns as well as the context.

#### Laparoscope motion patterns

A laparoscope motion pattern is defined as a sequence of movements before the laparoscope stops. A motion may contain a single command or several commands in a row. In this analysis, five types of motions can be observed:

- Single-decoupled motion: A motion only contains a decoupled command
- Single-coupled motion: A motion only contains a coupled command
- Multiple-decoupled motion: A motion contains multiple decoupled commands
- Multiple-coupled motion: A motion contains multiple coupled commands
- Mixed command motion: A motion contains decoupled and coupled commands

Performed motion patterns were compared between the experienced and trainee surgeon groups.

#### Context dependency

The context dependency concerns the task phases, the participants’ experience and the laparoscope moving speed.

Based on our observation, a complete surgery-like task is broken into two phases: the operation phase and the view transfer phase. The operation phase is when the participant focuses on hands-on work with few camera adjustments. The view transfer phase is defined as the period where the participant finishes one operation and moves to a new site. The view transfer phase usually requires the laparoscope to travel more than the operation phase. Every command sent from the FI was recorded, and the task phases were manually annotated.

In the PP task, the operation phase consists of two sub-phases: picking and placing an object from/to the corresponding poles. The view transfer phase is split into the object-holding phase moving towards a target pole (hereby shortened as object-holding phase) and the no-object phase moving towards the centre pole (hereby shortened as no-object phase). One complete pick and place operation starts from the no-object phase, then the picking phase, the object-holding phase and ends with the placing phase. The picking phase is considered easier than the placing phase since participants can finish picking as long as the target is visible in the view. In contrast, the placing phase needs a more clear view of the spatial relationship between a target pole and the holding object. Therefore, more frequent adjustments and larger motions are expected before the placing operation.

In the LT task, the operation phase starts when the thread enters the gate and stops when the needle exits the gate. The view transfer phase is the period after the needle leaves the current gate and before it enters the new gate.

Participants were split into an experienced surgeons group and a trainee surgeons group based on their experience. In actual surgeries, the experienced surgeons perform the hands-on operation, while the trainee surgeons control the laparoscope to assist them. Based on the real-life task assignment, we expect the trainee group to use more commands than the surgeon group due to their less experience in surgery.

Only the laparoscope angular speed of pure rotation was used in the analysis as the pure rotation takes 85% of the total motions. However, a motion like rotation with zooming out dramatically increases the user’s field of view, the rotation command only lasts for a very short time, usually less than a second. So any coupled command containing an insertion is excluded from the analysis due to insufficient valid recorded data.

### Safety

Patients’ safety is the first concern in any surgery. Four metrics listed below were used to evaluate the safety of the control:

- The number of times the object drops: Surgeons may inadvertently drop the object and leave it inside the patient body. This is dangerous in real surgery.
- The total time that the held object is outside of the laparoscope view: Keeping the held object outside the view is dangerous as the user cannot see the object, which decreases their situational awareness.
- The number of times the tool hits the board: in the setup, the baseboard is analogous to the patient’s tissue and the pole/gate is the operation site. Hitting the patient with the tools should be avoided.
- Participants were asked to identify the approach they find less error-prone if implemented in the actual surgery in questionnaires.

## Results

In this section, we first report the results of the target aiming task to evaluate the predictability and efficiency of the decoupled and hybrid control approaches, followed by laparoscope motion patterns, the context dependency, and safety of the laparoscope control in the PP and LT tasks. Although the sample size is small, statistical analyses is also presented. We use the Shapiro-Wilk method to check all data normality first. For paired samples comparison, such as if all eleven participants sent commands in decoupled and hybrid approaches, the paired t-test is used for normally distributed data and the Wilcoxon singed-rank test is used for non-normal distributed data. For independent samples comparison, such as the experienced surgeons and the trainee surgeons’ performance difference, we use pooled variances t-test for normally distributed data and the Mann-Whitney U test for non-normal distributed data. The significance level $\alpha$ = 0.05 is applied to all hypothesis tests. The statistical power is calculated using GPower 3.1.9.4. For the paired t-test, the statistical power is 0.32, the statistical power of Wilcoxon singed-rank test is 0.44, the pooled variances t-test’s statistical power is 0.11, and the Mann-Whitney U test’s power is 0.18.

### Assumptions validation

#### Predictability assumption validation

**Figure 4:**
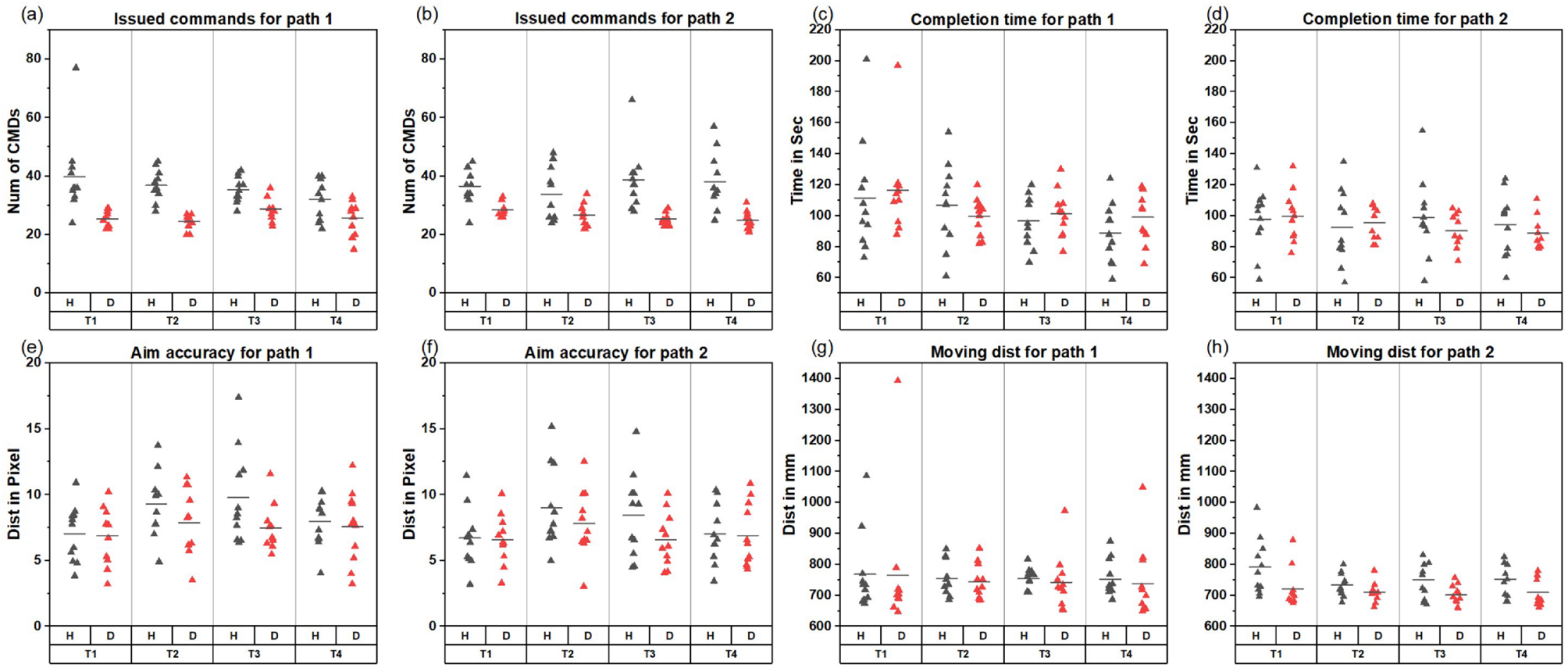
Participants’ performance in the target aiming task (**a**) Issued commands for path 1. (**b**) Issued commands for path 2. (**c**) Completion time for path 1. (**d**) Completion time for path 2. (**e**) Aiming accuracy for path 1. (**f**) Aiming accuracy for path 2. (**g**) Moving distance for path 1. (**h**) Moving distance for path 2. (T1 to T4 are the four trials for each path; H, D are hybrid and decoupled control). Average lines are the average measured metrics of eleven participating surgeons in each trial of every control method.

From Fig. 4a,b, decoupled control required fewer issued commands in all trials and two paths. The statistical analyses also show that decoupled control used significantly fewer commands. The maximum difference was in Trial 1 Path 1 (Decoupled 25.5 s vs Hybrid 39.8 s, *p* = 0.002), and the minimum difference happened in Trial 4 Path 1 (Decoupled 25.7 s vs Hybrid 32.1 s, *p* = 0.045), meaning it requires less adjustment compared to hybrid control. This result supports the assumption that predicting the movements of the end effector is easier using decoupled control.

#### Efficiency assumption validation

Efficiency is measured by the completion time, moving distance and accuracy.

In Fig. 4c,d, there was no obvious difference in task completion time between the two approaches and the analyses did not show any significant difference as well. The maximum time difference in Path 1 happened at Trail 4 (Decoupled 99.1 s vs Hybrid 88.2 s ), and that value for Path 2 was at Trial 3 (Decoupled 90.2 vs Hybrid 99). Sample points are more compact for decoupled control, which indicates that participants had a more consistent completion time with decoupled control.

The decoupled approach consistently had an equal to or better accuracy than the hybrid approach across both paths and all the trials in the aiming accuracy (Fig. 4e,f). Only one significant difference in Path 2 Trial 3 was identified (Decoupled 6.6 pixel vs Hybrid 8.5 pixel, *p* = 0.0297). Decoupled control moving distances were slightly shorter compared to hybrid control in all trials (Fig. 4g,h), and two significant differences were observed at Path 2 Trial 1 (Decoupled 720.5 mm vs Hybrid 791.2 mm, *p* = 0.00098) and Path 2 Trial 3 (Decoupled 701.9 mm vs Hybrid 750 mm, *p* = 0.0026).

In conclusion, decoupled control had overall shorter moving distances and higher accuracy. No obvious time difference was observed between the two approaches. Therefore, the assumption that hybrid control is more efficient than decoupled control is not supported.

#### Questionnaire

At the end of the target aiming task (Fig. 3a), the Comparative Control Strategy Questionnaire shows that 73% of participants considered the hybrid control more efficient than the decoupled control. In the predictability question, 55% of participants believed the decoupled method was more predictable. In addition, this questionnaire shows that 82% of participants identified hybrid control as a more distracting strategy. Only 18% of the participants considered the hybrid control to be less error-prone if implemented in actual surgery. Seven participants preferred using decoupled control.

Similarly, Task Feedback Questionnaires show participants believed decoupled gestures were more intuitive than hybrid foot gestures. The average score for decoupled control and hybrid control is 3.1 and 2.64 respectively. The average distraction score was a little bit higher for hybrid control (3.27) than decoupled control (2.91). Moreover, decoupled control (3.73) was considered to have better usability than hybrid control (3.31). However, no significant difference is found from the statistical analyses.

The NASA Task Load Index questionnaire shows hybrid control was rated higher in the weighted effort (12.4 vs 8.67, *p* = 0.1), mental demand (11.7 vs 6.24, *p* = 0.0039), and frustration level (7.52 vs 2.91, *p* = 0.16) than decoupled control, which means participants experienced more mental burden while using the hybrid approach. In terms of the physical aspect, the two methods had similar scores (Decoupled 6.6 vs Hybrid 7.4, *p* = 0.8). Decoupled control’s average overall perceived workload was significantly lower than hybrid control (Decoupled 38.53 vs Hybrid 51.67, *p* = 0.0037).

Overall, both predictability and efficiency comparison results from the questionnaires were the same as the assumptions we mentioned in the introduction section. In addition, decoupled control was reported to be potentially safer and less distracting from the Comparative Control Strategy Questionnaire and Task Feedback Questionnaires. Participating surgeons preferred using decoupled control as well. The NASA Task Load Index questionnaire also indicated that hybrid control caused a higher perceived workload than decoupled control, especially the cognitive load, and participants needed to make more effort to finish the target aiming task so they usually had a higher frustration level.

**Figure 5:**
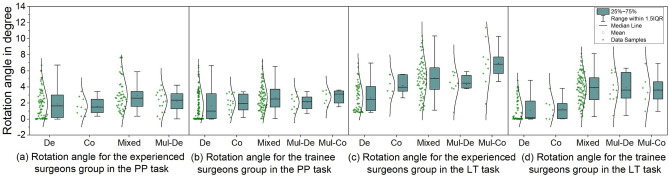
The laparoscope rotation angle of motions for the two groups in the Pick &Place (PP) and lead-through (LT) tasks. De, Co, Mul-De, Mul-Co and Mixed represent the Single-decoupled motion, Single-coupled motion, Multiple-decoupled motion, Multiple-coupled motion and Mixed command motion, respectively. Dots are all rotation angles of a motion pattern sent by eleven participating surgeons across all paths and trials. The curve and box plots show data distribution. Note that no experienced surgeon used Multiple-coupled motion in the PP task.

### Interface usage pattern

In this section, the findings of the laparoscope motion patterns are presented first, followed by the findings of the context dependency.

**Figure 6:**
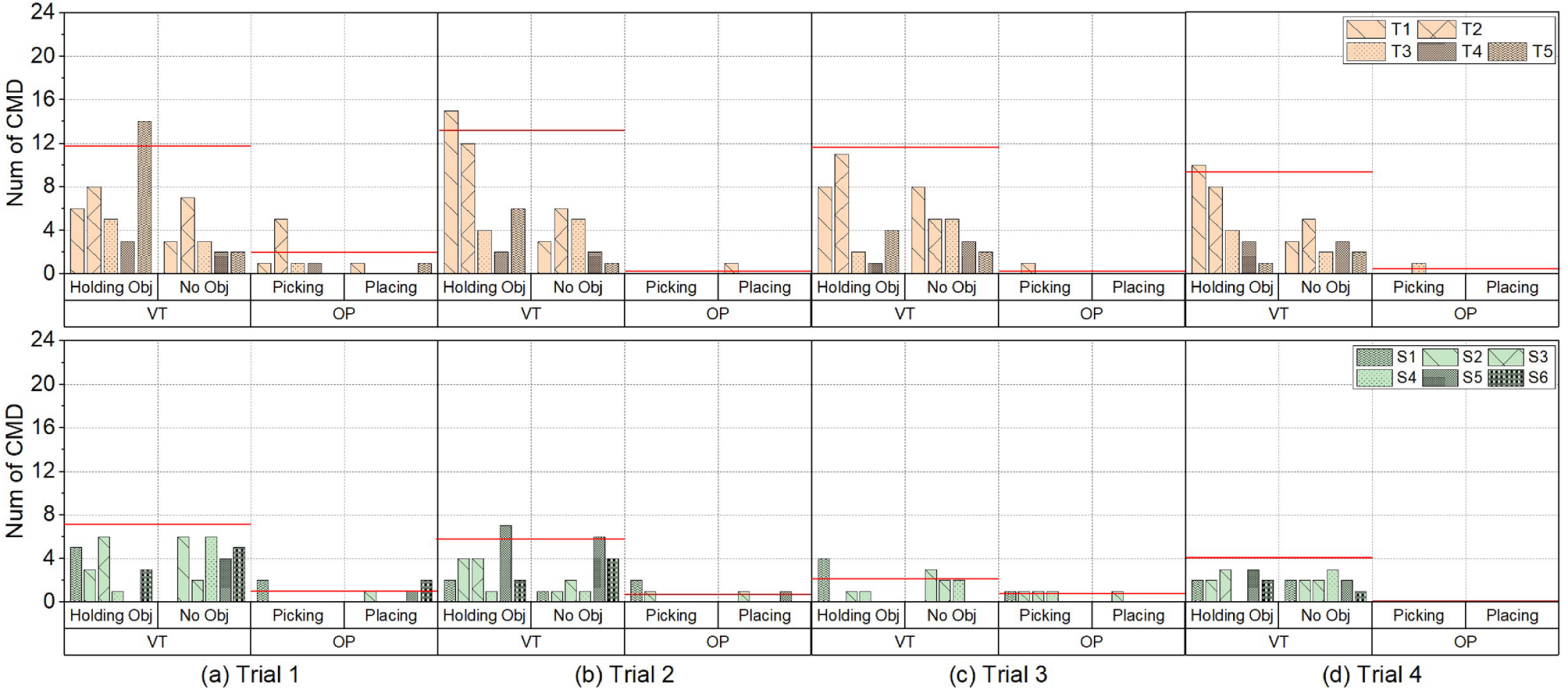
Total number of issued commands in the Pick &Place task for the trainee surgeons (light orange samples) and experienced surgeons (green samples). The four phases are grouped according to the task phases (VT is the view transfer phase and OP is the operation phase), while the correct sequence is a no-object phase, pick phase, object-holding phase and finally the placing phase. T1–T5 represent the five trainee surgeons and S1–S6 represent the six experienced surgeons. Red average lines are only used for visualisation purpose.

**Figure 7:**
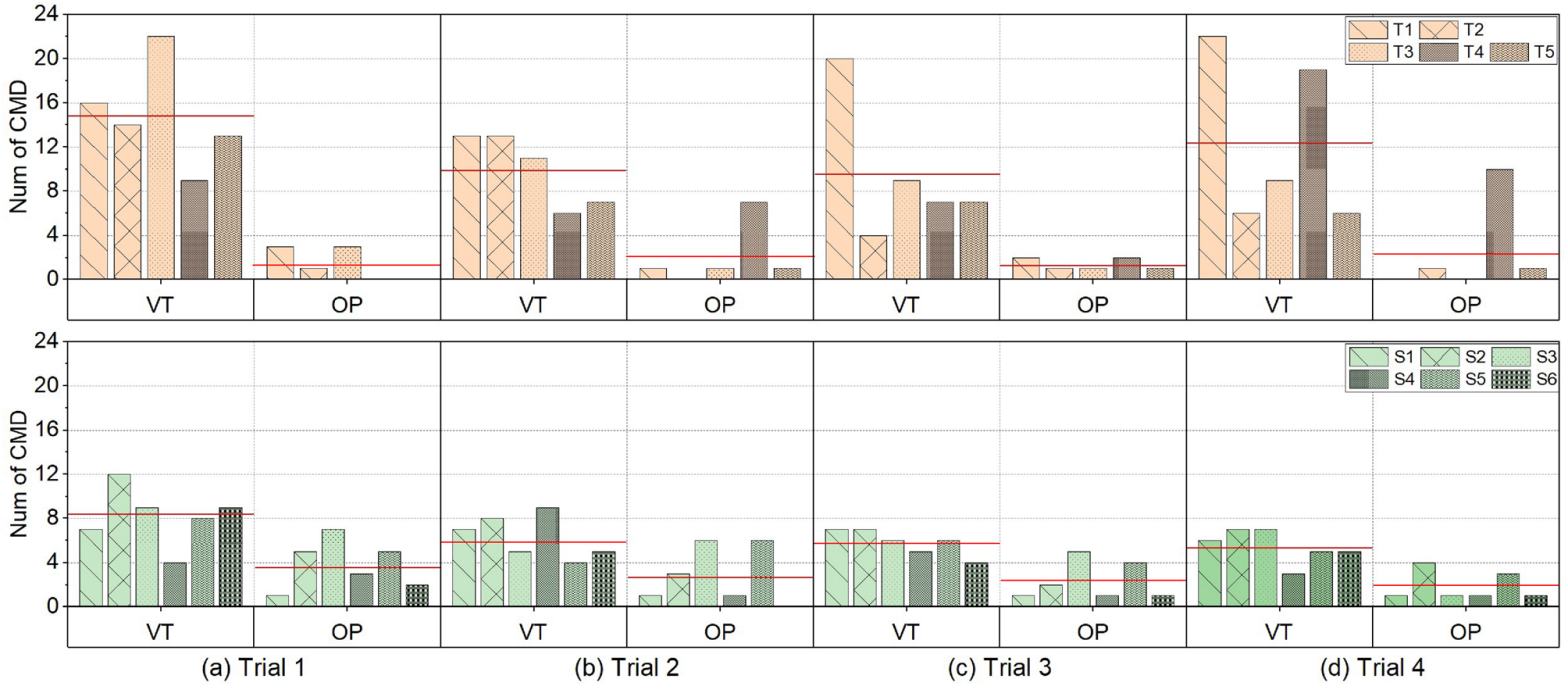
Total number of issued commands in the Lead-through task for the trainee surgeons and experienced surgeons. VT is the view transfer phase and OP is the operation phase. Red average lines are only used for the visualization purpose.

#### Laparoscope motion patterns

Across all paths and trials, 216 and 145 valid motions (camera travel angle larger than zero) were collected for the trainee surgeons and the experienced surgeons in the PP task. 193 and 147 valid motions were collected for the trainees and the experienced surgeons in the LT task (Fig. 5). The thresholds for small and large adjustments were defined as the first and third quarterlies, which were calculated based on each motion pattern in two surgery-like tasks for each surgeon group.

Based on the collected data and the video recordings, we observed two findings relate to laparoscope motion patterns among all the participants. (1) The Single-decoupled motion and the Mixed command motion were the two most frequently used motions among all participants in both surgery-like tasks. The Single-decoupled motion was frequently used in small laparoscope view adjustments and the Mixed command motion was commonly used in larger view adjustments. (2) It is also observed that in the Mixed command motion, a coupled commands was usually starting from a decoupled command and almost all three-DoF commands were actuated after a two-DoF command and rarely after a single axis command.

The Single-decoupled and the Mixed command motions had higher ratio than the rest of three motions in the two tasks. In the PP task, Single-decoupled command motions composed 47.7% and 50% of the total motions in the trainee and the experienced surgeons groups. The Mixed command motions occupied 38% and 33.8% of total motions for the trainee and the experienced surgeons groups. Comparably, in the LT task (Fig. 5c,d), the Single-decoupled motions ratio was 34.7% and 37.4% in the trainee and the experienced surgeons groups. The Mixed command motions ratio for the trainees and the experienced surgeon groups was 52.3% and 46.3% respectively.

Each of these two motions were found to be related to a corresponding distance-dependent use scenario in the two surgery-like tasks. In the PP task (Fig. 5a,b), the single-decoupled motion covered 72% and 85% of motions that were smaller than the minor adjustment threshold for the experienced and trainee surgeon groups. And these ratios were 73%, and 83% for the two groups in the LT task. In contrast, the Mixed command motion took 42% and 50% of motions that were larger than the greater view adjustment threshold for the trainee and the experienced surgeons groups in the PP task (Fig. 5a,b). These ratios were 70% and 85% for the two groups, respectively, in the LT task.

The command combination sequence is also identified to follow a specific pattern. 85.5% and 74% of Mixed command motions started with a decoupled command for the trainees and the experienced surgeons, respectively, in the PP task. 84.4% and 70% Mixed command motions started with a decoupled command for the trainees and the experienced surgeons, respectively, in the LT task. As for the three-DoF coupled command, only 116 were recorded after the whole experiment, and 82.8% of this command was actuated after a coupled command.

#### Context dependency

Due to the similarity of command use patterns in paths one and two, results are reported only for the shorter path in the PP task and the original setting for the LT task. Two findings were identified from the surgery-like tasks. (1) Both the experienced and trainee surgeons issued more commands in the view transfer phase than in the operation phase in both surgery-like tasks. Notably, the trainee surgeons had to make more frequent adjustments to the laparoscopic compared to the experienced counterparts in the view transfer stage. (2) At high speeds, both the experienced and trainee surgeons issued more coupled commands than decoupled commands in both pitch & yaw directions.

In the PP task (Fig. 6), most commands were sent in the view transfer phase. The ratio of commands sent in the view transfer phase to the total commands in four trials was 84.1%, 98.2%, 98%, and 97.6% for the trainee surgeons group, and 87.2%, 87.5%, 72.2%, and 100% for the experienced surgeons group. In the LT task, like the PP task, most commands were in the view transfer phase compared to the operation phase (Fig. 7), and the four trials’ ratios were 91.4%, 83.3%, 87%, and 83.8% for the trainee surgeons group, and 68%, 68.5%, 71.4%, and 75% for the experienced surgeons group. Across both tasks and their four trials, the average send command from the experienced surgeon group was lower than the number from the trainee group. In the LT task’s first two trials, The experienced group used significantly fewer commands than the trainee group (Trial 1: The experienced group 8.2 vs The trainee group 14.8, *p* = 0.017; Trial 2: The experienced group 6.3 vs The trainee group 10, *p* = 0.048). In the PP task’s last two trials, The experienced group used significantly fewer commands than the trainee group (Trial 3: The experienced group 2.2 vs The trainee group 9.8, *p* = 0.02; Trial 4: The experienced group 4 vs The trainee group 8.6, *p* = 0.003). So, we conclude the trainees used more commands than the experienced surgeons in the view transfer phase.

The trainee surgeons’ issued decoupled commands’ speed range of pitch & yaw concentrated between 0.032-0.036 rad/s and 0.034-0.043 rad/s across the two tasks, while the coupled pitch & yaw speed range was 0.04-0.05 rad/s. Similarly, The experienced surgeons’ issued decoupled commands’ speed range of pitch & yaw were between 0.032-0.04 rad/s and 0.035-0.045 rad/s, and the coupled speed range was higher in 0.038-0.065 rad/s.

### Safety

#### Dropping error

In the PP task, three experienced surgeons had dropping errors in the first path, and four surgeons had errors in the second path on average one dropping error in two trials. Four trainees had dropping errors in the first path, and all trainees had these errors in the second path. Almost all trainees experienced at least one dropping error in a single trial of both paths. The maximum number of dropping errors of the two groups in one trial was three. In the LT task, only one experienced surgeon had a dropping error in two trials. One trainee had dropping errors in each path. Recordings show that almost all dropping errors happened when the participants transferred the object or the needle with the laparoscope either static or moving slowly.

#### Outside-the-view error

In the PP task, only one trainee had an outside-the-view error in the shorter path, while two experienced surgeons made this error in the shorter path, and one experienced surgeon also had this issue in the longer path. In the LT task, all five trainees had at least one outside-the-view error in the original and the mirrored settings. Four of the six experienced surgeons made at least one of this type of error in the original setting. Similarly, four experienced surgeons made at least one of this type of error in the mirrored setting. These four experienced surgeons in the two settings were not the same, which means all six experienced surgeons made at least one outside-the-view error in the LT task.

#### Hitting error

No hitting error was observed among all the participants in the PP and LT tasks.

## Discussion

### Assumptions validation

The assumption that decoupled control makes it easier to predict the final orientation of the end effector was supported as it required fewer issued commands, implying fewer adjustments or corrections. The questionnaires confirmed that most participants perceived that predictability was better for decoupled control, consistent with the experiment result. On the other hand, based on the objective measures of performance, the assumption that hybrid control is more efficient compared to decoupled control was not supported. However, the questionnaires contradicted this result. This indicates that although the user might perceive a higher efficiency using hybrid control, this does not necessarily translate into a better performance.

### Interface usage patterns

#### Laparoscope motion patterns

In this section, we discuss the two consistent usage patterns in the two surgery-like tasks. The Singled-decoupled motion was observed as a commonly used motion pattern for minor view adjustments and the Mixed command motion was a popular motion pattern in larger view adjustments.

Upon reviewing the video footage, we found that smaller laparoscope adjustments can be normally interpreted as delicate moves in the operation phase or limited laparoscope adjustment in the view transfer phase, i.e., bringing the target into the view. One of the possible reasons for the frequent use of the Single-decoupled motion in the operation phase is the decoupled control was assessed as less distracting and caused low cognitive load. Thus, the participants could focus on demanding hands-on tasks if any adjustment of the view during the operation was necessary. It also relates to workload management. We also observed that the Mixed command motion was usually bundled with larger view adjustments. we infer this phenomenon is based on a misconception between efficiency and hybrid control. The results from the laparoscope manipulation tasks show similar time efficiency between the two control strategies. However, hybrid control was usually considered to be faster and more efficient than decoupled control in questionnaires. In the two surgery-like tasks, participants were informed that the completion time is one of the evaluation metrics. Thus, when larger view adjustments were required, most participants tended to use coupled commands to save time. Because over 95% coupled commands belonged to the Mixed command motion, we relate the Mixed command motion to larger view adjustment.

Although the Mixed command motion contains decoupled and coupled commands, decoupled commands were usually used at the beginning of the motion and only lasted a very short time. one possible reason that the motion usually started from a decoupled command could be that participants were confident in using decoupled control as the target aiming task shows that this method had better predictability. Based on the perceived laparoscope moving status under the decoupled motion, it should be easier for them to choose a proper coupled command speed. So when a participant wants to issue a coupled command, they usually use the Mixed command motion instead of the Single-coupled or Multiple-coupled motions. This also explains why almost all three-DoF commands were actuated after a two-DoF command and rarely after a single-axis command, as the participants gradually built a complex command.

#### Context dependency

The context dependency analysis considers the commands issued by all participating surgeons in different task phases and the commands speed to study how participating surgeons manage their workload in the two surgery-like tasks while maintaining a good performance.

Both two groups issued more commands in the view transfer phase than in the operation phase. The most likely reason is that controlling a laparoscope is a new secondary task compared to the primary surgical procedure using new technology: the FI, demanding additional cognitive load^6^. Thus, participants usually adjust their laparoscopic view before any complex hands-on operation to focus on their hands-on tasks and avoid distraction. Moreover, in both surgery-like tasks, the trainees issued more commands in the view transfer phase than the experienced surgeons. One potential reason could be that, in surgeries, trainees are usually responsible for laparoscope control and have less chance to operate. Therefore, they tend to bring the target to the centre of the view for easier operations. In contrast, experienced surgeons usually started to operate as soon as the target became visible in the laparoscopic view. Another potential reason could be the difference in the zoom control logic between the two groups. The trainee surgeons usually moved directly to the next target without considering the field of view. However, the experienced surgeons usually zoomed out first to gain a broader view to locate a new target site with fewer view adjustments. Therefore, reducing the number of issued commands. In the operation phase, commands were limited since all participants only made limited adjustments, and thus we did not make any conclusions in that phase. In Figs. 6 and 7, the high command numbers in this phase are often due to trainees issuing more commands to bring the target to the centre of the view, or the participant using extra commands to search for the dropped object outside the view.

The increased speed of coupled commands, in comparison to decoupled commands, is also connected to the aforementioned misconception regarding efficiency and hybrid control. Participants frequently opted for coupled commands at higher speeds to expedite larger view adjustments to save time.

### Safety

The dropping error was higher in the PP task than in the LT task since grasping was easier with the soft needle used in the LT task. Thus, fewer errors were observed. There were more outside-the-view errors in the LT task since the gates were further apart compared to the pole distance in the PP task. Thus, some participants would move the needle close to the gate quickly before the gate was in the view. However, almost all participants forgot to stop the laparoscope when they were focusing on the hands-on task until the laparoscope hit the virtual boundary or the operation site was not visible in the view. The first situation did not cause any problems because the laparoscope stopped automatically, and the participant could continually focus on the current task. If the operation site was out of view, the participant needed to issue an additional decoupled command to bring the site back to the centre of the view since the distance was usually not far.

### Foot interface guideline

Based on the participants’ feedback, the FI usage patterns and safety concerns, two FI design suggestions for the endoscope manipulation task are provided: (1) Decoupled control is more suitable for the endoscope control task compared to the hybrid control. Results show that the decoupled control has better predictability and similar efficiency, while decreasing the surgeon’s cognitive load. Although participants believed that the hybrid approach was more intuitive and efficient, it might be a misperception since they commonly actuated coupled commands at higher speeds. (2) A graphical indicator on the laparoscopic view indicating laparoscope movement would be helpful in avoiding unintentional actuation, i.e., the “laparoscope is moving” warning disappearing from the screen only when the laparoscope stops.

We expect similar usage patterns for the endoscope control in other types of tasks as they require a similar set of skills as the ones studied in this work. In other robot-assisted surgical activities, such as surgical tool manipulation, if hybrid control is required, designers could consider removing the three-DoF command as it requires more cognitive skills than other commands and was rarely used. It could also reduce surgeons’ cognitive load as there will be fewer commands to remember. Overall, a context-aware system that enables only the decoupled control in the operation phase might improve the operation’s accuracy and safety.

## Conclusion

This paper first compares the decoupled and hybrid approaches during standalone laparoscope control to validate the assumptions about efficiency and predictability. It then uses two bimanual surgery-like tasks to study the FI usage pattern and safety when choosing among different laparoscope motion patterns in laparoscope control, showing the decoupled control is more suitable for the laparoscope control task. Findings contribute to the FI design of the laparoscope control task in RA-MIS as well as other Human-Robot collaboration or teleportation tasks in surgical and industrial applications.

The end users’ feedback is critical in the development of such an interface for human-robot collaborations. The user’s additional cognitive load caused by the new task should be limited through the interface design and control algorithm for improved safety and efficiency.

As the first study to directly compare the decoupled and hybrid control strategies using a laparoscope control task, results show that both approaches have similar efficiency and decoupled control has better predictability. The interface usage pattern study results show the typical scenario for choosing the motion pattern and provide an essential guideline for FI control strategy selection. These results illustrate the participating surgeons’ preferences and usage while they are initially adapting to the interface.

Our paper has some limitations. The main limitation is the small sample size. Even if we do the statistical analysis, the statistical power is low, and all the power numbers are lower than 0.8. To make sure the test detects true effects, we need to recruit at least 64 experienced surgeons and 64 trainee surgeons. In addition, the participants’ mastery of using the FI was not tested after the 5-minute training session, mainly due to the time limitations. The participating surgeon’s fatigue was only reported based on their feedback and questionnaire results. More common fatigue measurement methods such as EMG techniques and Borg scale should be implemented in the future. Furthermore, this experiment assessed the participating surgeons’ performance when they used the new interface for the first time. It is not clear if these results would remain consistent with extended use and in a more time-consuming surgery-like task. Therefore, future work should conduct larger and longer-duration experiments to address these limitations. Last, only visual feedback about the FI movements is provided to the users. Another additional or different feedback channel may have a different effect on the surgeon’s performance.

Although the results focus on the FI design in the laparoscope control research, the experiment protocol sets the stage and can be modified for other human-robot interaction studies, especially the studies that apply tool-manipulating robotic arms in surgical and industry applications. This kind of research usually requires good user-tool coordination performance in addition to controllability. For example, the surgeon may need to use a robotic arm to move an additional assistive tool to retract an organ for their cutting operation. Because the protocol combines basic and simulation tasks, it can comprehensively evaluate both the user’s essential manipulation performance and human-tool coordination skills. Moreover, observing users’ usage patterns is also critical because it can be used to study their implicit needs that are not shown in the design requirements.

### Supplementary Information

Supplementary Information 1.

Supplementary Information 2.

Supplementary Information 3.

Supplementary Information 4.

Supplementary Information 5.

Supplementary Information 6.

Supplementary Information 7.

## Data Availability

The data that support the findings of this study are available from RoMI Lab at Monash University but restrictions apply to the availability of these data. Researchers are welcome to contact elahe.abdi@monash for more information.
