# Supplementary material for: Experimental evaluation of accuracy and efficiency of two control strategies for a novel foot commanded robotic laparoscope holders with surgeons
Source: Sci Rep. 2024 Apr 23;14:9264. doi: 10.1038/s41598-024-59338-3 (PMC11035708; doi:10.1038/s41598-024-59338-3)
Supplement: Supplementary file 1 — Supplementary Information 1. [file 41598_2024_59338_MOESM1_ESM.pdf]

Pure laparoscope manipulation task

1. It was easy to finish the task quickly using this control strategy.

	1	2	3	4	5	
**Strongly Disagree**	☐	☐	☐	☐	☐	**Strongly Agree**

2. Using coupled commands helps me finish this task faster.

	1	2	3	4	5	
**Strongly Disagree**	☐	☐	☐	☐	☐	**Strongly Agree**

3. I always needed to recall what gesture to do before conducting commands.

	1	2	3	4	5	
**Strongly Disagree**	☐	☐	☐	☐	☐	**Strongly Agree**

4. I always needed to watch the screen and check the foot gesture.

	1	2	3	4	5	
**Strongly Disagree**	☐	☐	☐	☐	☐	**Strongly Agree**

5. I could learn this control strategy fast.

	1	2	3	4	5	
**Strongly Disagree**	☐	☐	☐	☐	☐	**Strongly Agree**

6. I managed to use this control strategy well.

	1	2	3	4	5	
**Strongly Disagree**	☐	☐	☐	☐	☐	**Strongly Agree**

7. All the combination commands are necessary for the control.

	1	2	3	4	5	
**Strongly Disagree**	☐	☐	☐	☐	☐	**Strongly Agree**

8. I felt physically tired after the experiment.

	1	2	3	4	5	
**Strongly Disagree**	☐	☐	☐	☐	☐	**Strongly Agree**

9. I felt mentally tired after the experiment.

	1	2	3	4	5	
**Strongly Disagree**	☐	☐	☐	☐	☐	**Strongly Agree**

Surgery-like task

1. Removing coupled commands, relying on only decoupled commands will significantly affect my performance.

	1	2	3	4	5	
**Strongly Disagree**	☐	☐	☐	☐	☐	**Strongly Agree**

2. How frequently did you use the coupled commands in this task?

	1	2	3	4	5	
**Rarely use**	☐	☐	☐	☐	☐	**Frequently use**

3. Please sort the following commands type according to your use frequency (1 is lowest and 5 is highest).

	1	2	3	4
**Decoupled command**	☐	☐	☐	☐
**Single translation+zoom**	☐	☐	☐	☐
**Dual translation command**	☐	☐	☐	☐
**Dual translation+zoom**	☐	☐	☐	☐

4. I always needed to recall what gesture to do before conducting coupled command.

	1	2	3	4	5	
**Strongly Disagree**	☐	☐	☐	☐	☐	**Strongly Agree**

5. I always needed to recall what gesture to do before conducting decoupled command.

	1	2	3	4	5	
**Strongly Disagree**	☐	☐	☐	☐	☐	**Strongly Agree**

6. I always needed to watch the screen and check the foot gesture when conducting the coupled command.

	1	2	3	4	5	
**Strongly Disagree**	☐	☐	☐	☐	☐	**Strongly Agree**

7. I always needed to watch the screen and check the foot gesture when conducting the decoupled command.

	1	2	3	4	5	
**Strongly Disagree**	☐	☐	☐	☐	☐	**Strongly Agree**
