# Supplementary material for: Experimental evaluation of accuracy and efficiency of two control strategies for a novel foot commanded robotic laparoscope holders with surgeons
Source: Sci Rep. 2024 Apr 23;14:9264. doi: 10.1038/s41598-024-59338-3 (PMC11035708; doi:10.1038/s41598-024-59338-3)
Supplement: Supplementary file 2 — Supplementary Information 2. [file 41598_2024_59338_MOESM2_ESM.pdf]

1. Please select the option closest to your job title.

☐ **Surgeon** ☐ **Registrar / Fellow**

2. Do you have experience with foot interfaces?

☐ **Never** ☐ **Less than fifty times** ☐ **More than fifty times**

3. Do you have experience with Fundamentals of Laparoscopic Surgery (FLS)?

☐ **Never** ☐ **Sometimes** ☐ **Often**

4. Do you have experience with minimally invasive surgery?

☐ **Never** ☐ **Less than fifty times** ☐ **More than fifty times**

5. Do you have experience with robotic-assisted minimally invasive surgery?

☐ **Never** ☐ **Less than fifty times** ☐ **More than fifty times**

6. Please select your dominant leg.

☐ **Left leg** ☐ **Right leg**

7. Which control strategy was more efficient to use?

☐ **Hybrid control** ☐ **Decoupled control** ☐ **Neither** ☐ **Both**

8. Which control strategy was more distracting to use?

☐ **Hybrid control** ☐ **Decoupled control** ☐ **Neither** ☐ **Both**

9. Which control strategy was more intuitive to use?

☐ **Hybrid control** ☐ **Decoupled control** ☐ **Neither** ☐ **Both**

10. Which control strategy will be less error-prone if implemented in the actual surgery?

☐ **Hybrid control** ☐ **Decoupled control** ☐ **Neither** ☐ **Both**

11. Which control strategy was safer to aim at a target?

☐ **Hybrid control** ☐ **Decoupled control** ☐ **Neither** ☐ **Both**

12. Which control strategy was mentally more tiring for you?

☐ **Hybrid control** ☐ **Decoupled control** ☐ **Neither** ☐ **Both**

13. Which control strategy was physically more tiring for you?

☐ **Hybrid control** ☐ **Decoupled control** ☐ **Neither** ☐ **Both**

14. Which control strategy did you prefer to use to finish the tasks?

☐ **Hybrid control** ☐ **Decoupled control** ☐ **Neither** ☐ **Both**

15. Why you prefer using the selected control strategy?

____

16. Using which control strategy did you feel more in control?

☐ **Hybrid control** ☐ **Decoupled control**

17. Comments to the coupled and decoupled control strategies?

____
