# Supplementary material for: Experimental evaluation of accuracy and efficiency of two control strategies for a novel foot commanded robotic laparoscope holders with surgeons
Source: Sci Rep. 2024 Apr 23;14:9264. doi: 10.1038/s41598-024-59338-3 (PMC11035708; doi:10.1038/s41598-024-59338-3)
Supplement: Supplementary file 3 — Supplementary Information 3. [file 41598_2024_59338_MOESM3_ESM.pdf]

Pure laparoscope manipulation task

1. It was easy to finish the task quickly using this control strategy.

	1	2	3	4	5	
**Strongly Disagree**	☐	☐	☐	☐	☐	**Strongly Agree**

2. I always needed to recall what gesture to do before conducting commands.

	1	2	3	4	5	
**Strongly Disagree**	☐	☐	☐	☐	☐	**Strongly Agree**

3. I always needed to watch the screen and check the foot gesture.

	1	2	3	4	5	
**Strongly Disagree**	☐	☐	☐	☐	☐	**Strongly Agree**

4. I could learn this control strategy fast.

	1	2	3	4	5	
**Strongly Disagree**	☐	☐	☐	☐	☐	**Strongly Agree**

5. I managed to use this control strategy well.

	1	2	3	4	5	
**Strongly Disagree**	☐	☐	☐	☐	☐	**Strongly Agree**

6. I felt physically tired after the experiment.

	1	2	3	4	5	
**Strongly Disagree**	☐	☐	☐	☐	☐	**Strongly Agree**

7. I felt mentally tired after the experiment.

	1	2	3	4	5	
**Strongly Disagree**	☐	☐	☐	☐	☐	**Strongly Agree**
