# Supplementary material for: Experimental evaluation of accuracy and efficiency of two control strategies for a novel foot commanded robotic laparoscope holders with surgeons
Source: Sci Rep. 2024 Apr 23;14:9264. doi: 10.1038/s41598-024-59338-3 (PMC11035708; doi:10.1038/s41598-024-59338-3)
Supplement: Supplementary file 5 — Supplementary Information 5. [file 41598_2024_59338_MOESM5_ESM.docx]

**Experiment Feedback Questionnaire - Decoupled.pdf**

This is a customized task feedback questionnaire for decoupled control. It was issued to every participant after they finished the target aiming task using decoupled control. It was used to assess the intuitiveness, usability and distraction of decoupled control.

**Experiment Feedback Questionnaire - Hybrid.pdf**

This is a customized task feedback questionnaire for hybrid control. It was issued to every participant after they finished the target aiming task using hybrid control. It was used to assess the intuitiveness, usability and distraction of hybrid control.

**Comparative Control strategy Questionnaire.pdf**

This questionnaire was issued to every participant after they finished the target aiming task using both control methods. It compares the two strategies’ predictability and efficiency in the target aiming task using single-choice questions.

**The video of foot command mapping and the three tasks.mp4**

The supplemented video features a demonstration of decoupled and hybrid control, showcasing a complete operation involving three tasks. The video initially exhibits one Degree of Freedom (DoF) foot gestures for both decoupled and hybrid control, followed by demonstrations of two DoF and three DoF foot gestures. During the presentation of the target aiming task, visual cues guide participants on laparoscope movement. Subsequently, two surgery-like tasks are recorded. All phases of both tasks are highlighted in the recordings for clear demonstration.
