# Supplementary material for: Experimental evaluation of accuracy and efficiency of two control strategies for a novel foot commanded robotic laparoscope holders with surgeons
Source: Sci Rep. 2024 Apr 23;14:9264. doi: 10.1038/s41598-024-59338-3 (PMC11035708; doi:10.1038/s41598-024-59338-3)
Supplement: Supplementary file 6 — Supplementary Information 6. [file 41598_2024_59338_MOESM6_ESM.docx]

| **Dropping error in the PP task** | | | |
| --- | --- | --- | --- |
|  | **First/Shorter path** | **Second/Longer path** | **Total** |
| **Experienced surgeon 1** | **3** | **0** | **3** |
| **Experienced surgeon 2** | **0** | **2** | **2** |
| **Experienced surgeon 3** | **0** | **0** | **0** |
| **Experienced surgeon 4** | **0** | **1** | **1** |
| **Experienced surgeon 5** | **6** | **4** | **10** |
| **Experienced surgeon 6** | **2** | **5** | **7** |
|  |  |  |  |
| **Trainee surgeon 1** | **1** | **4** | **5** |
| **Trainee surgeon 2** | **2** | **1** | **3** |
| **Trainee surgeon 3** | **0** | **2** | **2** |
| **Trainee surgeon 4** | **2** | **1** | **3** |
| **Trainee surgeon 5** | **1** | **1** | **2** |

**Supplementary table S1 Dropping error in the PP task.** This table provides the number of dropping error for each participant in all paths and trials in the pick and place task.

| **Outside-the-view error in the PP task** | | | |
| --- | --- | --- | --- |
|  | **First/Shorter path** | **Second/Longer path** | **Total** |
| **Experienced surgeon 1** | **11** | **0** | **11** |
| **Experienced surgeon 2** | **0** | **0** | **0** |
| **Experienced surgeon 3** | **0** | **5** | **5** |
| **Experienced surgeon 4** | **0** | **0** | **0** |
| **Experienced surgeon 5** | **2** | **0** | **2** |
| **Experienced surgeon 6** | **0** | **0** | **0** |
|  |  |  |  |
| **Trainee surgeon 1** | **0** | **0** | **0** |
| **Trainee surgeon 2** | **0** | **0** | **0** |
| **Trainee surgeon 3** | **0** | **0** | **0** |
| **Trainee surgeon 4** | **0** | **0** | **0** |
| **Trainee surgeon 5** | **6** | **0** | **6** |

**Supplementary table S2 Outside view error in the PP task.** This table provides the number of outside view error for each participant in all paths and trials in the pick and place task.

| **Dropping error in the LT task** | | | |
| --- | --- | --- | --- |
|  | **Original setup** | **Mirrored setup** | **Total** |
| **Experienced surgeon 1** | **0** | **3** | **3** |
| **Experienced surgeon 2** | **0** | **0** | **0** |
| **Experienced surgeon 3** | **0** | **0** | **0** |
| **Experienced surgeon 4** | **0** | **0** | **0** |
| **Experienced surgeon 5** | **0** | **0** | **0** |
| **Experienced surgeon 6** | **0** | **0** | **0** |
|  |  |  |  |
| **Trainee surgeon 1** | **0** | **0** | **0** |
| **Trainee surgeon 2** | **0** | **0** | **0** |
| **Trainee surgeon 3** | **0** | **0** | **0** |
| **Trainee surgeon 4** | **4** | **3** | **7** |
| **Trainee surgeon 5** | **0** | **0** | **0** |

**Supplementary table S3 Dropping error in the LT task.** This table provides the number of dropping error for each participant in all paths and trials in the lead-through task.

| **Outside-the-view error in the LT task** | | | |
| --- | --- | --- | --- |
|  | **Original setup** | **Mirrored setup** | **Total** |
| **Experienced surgeon 1** | **0** | **24** | **24** |
| **Experienced surgeon 2** | **5** | **0** | **5** |
| **Experienced surgeon 3** | **6** | **26** | **32** |
| **Experienced surgeon 4** | **9** | **2** | **11** |
| **Experienced surgeon 5** | **5** | **0** | **5** |
| **Experienced surgeon 6** | **0** | **2** | **2** |
|  |  |  |  |
| **Trainee surgeon 1** | **9** | **15** | **24** |
| **Trainee surgeon 2** | **4** | **4** | **8** |
| **Trainee surgeon 3** | **6** | **2** | **8** |
| **Trainee surgeon 4** | **16** | **8** | **24** |
| **Trainee surgeon 5** | **5** | **14** | **19** |

**Supplementary table S4 Outside view error in the LT task.** This table provides the number of outside view error for each participant in all paths and trials in the lead-through task.
